# The Effect of Aqueous Extract of *Glycyrrhiza glabra *on *Herpes Simplex Virus* *1*

**DOI:** 10.5812/jjm.11616

**Published:** 2014-07-01

**Authors:** Masoud Sabouri Ghannad, Avid Mohammadi, Sohayla Safiallahy, Javad Faradmal, Mona Azizi, Zohreh Ahmadvand

**Affiliations:** 1 Research Center for Molecular Medicine, Department of Microbiology, Faculty of Medicine, Hamadan University of Medical Sciences, Hamadan, IR Iran; 2 Department of Biostatistics and Epidemiology, School of Public Health, Hamadan University of Medical Sciences, Hamadan, IR Iran

**Keywords:** Herpesvirus 1, Human, *Glycyrrhiza glabra*, Glycyrrhetinic Acid, Antiviral Agents

## Abstract

**Background:**

*Herpes Simplex Virus 1* (HSV-1) resistance to drugs and the side effects of drugs have drawn the attention of investigators to herbal plants.

**Objectives:**

The main aim of the current research was to investigate the effects of *Glycyrrhiza glabra* (liquorice root) on HSV-1. One of the objectives of the current research was to determine the efficacy and the effect of the elapsed incubation time of treating the Vero cells infected with HSV-1 by *G. glabra*. In addition, the effect of cells pretreatment with licorice root extract, preincubation of virus with licorice root extract, and the antiviral activity were assessed.

**Patients and Methods:**

Vero cells were incubated after adding different concentrations of aqueous extracts of *G. glabra*. The cells were incubated during various time courses. Cytotoxicity assay, determining the 50% tissue culture infectious dose (TCID_50_), and incubation of HSV-1 with licorice root extract prior to viral infection were performed.

**Results:**

Internal association among different experiment groups showed the significant difference in the efficacy of the extract with regard to incubation period between one and four hours, one and eight hours, four and 12 hours, and eight and 12 hours. Moreover, there was a significant difference with regard to efficacy among the pretreatment of cells with extract for two hours, incubation of virus with extract for one hour, incubation of virus with extract for two hours.

**Conclusions:**

*G. glabra* showed the characteristics of a novel antiviral medication; however, more in vitro experiments are needed to determine the antiherpetic activities of the *G. glabra*.

## 1. Background

*Herpes simplex virus 1* (HSV-1), as a member of *Herpesviridae* can cause severe diseases in the neonates, the elderly, patients with drug-induced immunosuppression, and in those with acquired immunodeficiency syndrome (AIDS). Nowadays, increasing resistance to antiherpetic drugs are reported frequently (1). Due to the drug side effects and HSV resistance to antiviral drugs, especially resistance to acyclovir in high-risk immunocompromised patients, new medications including herbal plants such as *Glycyrrhiza glabra* (licorice root) have drawn especial interest. This highlights the need for new efficient and safe agents for treating HSV (2). Manuscripts from China, Greece, and India confirm the historical background of the *Glycyrrhiza* species use (3). In China, licorice (Gan Cao) has been reported as one of the oldest and most commonly prescribed traditional medicine, which has been used in the treatment of several diseases (4). *G. glabra* natural habitat are Southwest and Central Asia as well as subtropical and temperate areas of the planet, including Europe. The root is termed licorice and has a sweet odor and smell The genus *Glycyrrhiza* (*Leguminosae*) includes about 30 species such as *G.* *glabra*, *G.* *uralensis*, *G.* *inflata*, *G. aspera*, and Persian and Turkish licorices, which are determined as *G. glabra* var. *violace*.

The first report that indicated an antiviral property of licorice constituents dates back to 1979 (5). In that research, the scientists recognized glycyrrhizic acid and its antiviral activity in vitro, which suppressed the growth and cytopathic effects (CPE) of numerous DNA and RNA viruses, such as HSV-1, Newcastle disease, *Vaccinia virus*, and *vesicular stomatitis virus* (3). Some reports indicated that a few minor constituents of *G. glabra* such as liquiritigenin and isoliquiritigenin might have some pharmacological functions (4). Acyclovir, a nucleoside analog and an antiherpetic drug, has partially fulfilled the need for treating the infection entirely; however, it leads to viral resistance and consequently, viral latency and recurrence (6). Regarding the increasing rate of HSV resistance to drugs and the evidences of HSV-1 infection in the majority of women before fertility age in Iran (7) finding a novel anti-HSV drugs seems necessary (8).

## 2. Objectives

The main goal of the current study was in vitro evaluation of possible antiherpetic and virucidal activity of *G. glabra*. The possible virus inhibition yield under the effect of licorice extract was determined as 50% tissue culture infectious dose (TCID_50_) per milliliter in Vero cells. One of the objectives of the current research was to determine the efficacy with respect to the elapsed incubation time and treatment of the cells infected with HSV with the licorice extract to assess the antiviral activity of *G. glabra* in virus adsorption. In addition, the effect of pretreatment of cells with licorice root extract and preincubation of virus with *G. glabra* were other objectives of the current study.

## 3. Materials and Methods

### 3.1. Plant Material

Licorice root was prepared from Kermanshah, a western province of Iran, which has shown to have the licorice root of the best quality in Iran (9). Licorice roots were dried and grinned into powder. The powder was used for extraction. Four grams of dried powder was suspended in 100 mL sterile distilled water and kept in 37℃ for 24 hours and then incubated for eight hours in room temperature while being mixed by magnetic mixer. In the next step, the suspension was kept 18 hours at room temperature (9). The final mixture was passed through 0.45 µL filter and preserved at 4℃ until the time of use.

### 3.2. Cells and Virus

Vero cells (African green monkey kidney cells) were provided by the Department of Virology, Faculty of Public Health, Tehran University of Medical Sciences, Tehran, Iran. Vero cells were cultured with Dulbecco's Modified Eagle Medium (DMEM) supplemented with 10% fetal bovine serum (FBS) and addition of antibiotics, namely, penicillin and streptomycin. Vero cells were incubated at 37℃ with 5% CO_2_. *Herpes simplex virus 1* was obtained from Pasteur Institute, Tehran, Iran. The cytopathogenic dose of the HSV-1 was assessed and expressed as TCID_50_/ml (10).

### 3.3. Cytotoxicity Assay

**Figure 1.**
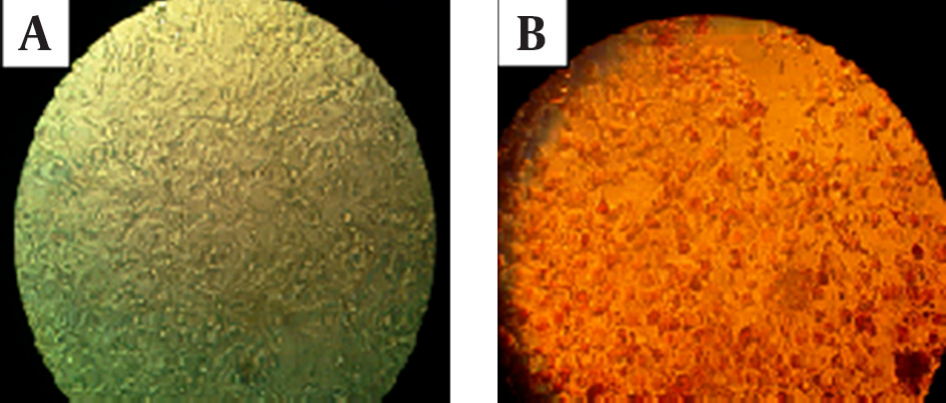
Staining the Vero Cells. A, Vero cells before staining with Neutral Red dye. B, Vero cells after treating with *G. glabra* and staining with Neutral Red dye.

In order to determine the appropriate concentration of aqueous extract of licorice root, which has less than 50% cytotoxicity for Vero cells, the Neutral Red assay was employed. Microtitre plates containing 96-well tissue culture plates were inoculated with 105 Vero cells to achieve the confluence of 80%. The cells were washed with pre-warmed phosphate buffered saline (PBS). Then different concentrations of licorice root extract prepared in DMEM were added to the assigned wells. The plates were incubated at 37℃ with 5% CO_2_ for two days. After two days, the Vero cells were washed by pre-warmed PBS and filtered Neutral Red solution was added to each well (The viable cells absorb the Neutral Red dye). The plates were incubated in CO_2_ incubator at 37℃ for three hours. After incubation, the cells were washed by pre-warmed PBS and Neutral Red dye stain solution was added to each well. The plates were shaken in dark on the shaker for ten minutes until the Neutral Red dye was removed. The optical density of the Neutral Red solution was measured at 540 nm by ELISA reader (Sunrise Remote, Tecan, Austria) (Table 1). The highest concentration that had less than 50% cytotoxicity for Vero cells (0.2 mg/mL) was used for further experiments (Figure 1).

**Table 1.** The Optical Density of Different Dilution Proportion of the Aqueous Licorice Root Extract in Cultured Cells to Determine the 50% Tissue Culture Infectious Dose for Vero Cells

	Blank	Cell Control	Dilution Proportion 1:10	Dilution Proportion 1:50	Dilution Proportion 1:100	Dilution Proportion 1:200	Dilution Proportion 1:400	Dilution Proportion 1:600	Dilution Proportion 1:800	Dilution Proportion 1:1000	Cell Control	Blank
**OD**	0.085	0.083	0.084	0.082	0.097	0.091	0.082	0.083	0.083	0.085	0.097	0.089
**OD**	0.091	0.458	0.084	0.311	0.525	0.554	0.498	0.5	0.498	0.467	0.52	0.096
**OD**	0.098	0.468	0.094	0.302	0.521	0.53	0.521	0.479	0.503	0.485	0.526	0.089
**OD**	0.086	0.404	0.096	0.325	0.509	0.542	0.505	0.537	0.489	0.459	0.485	0.091
**OD**	0.088	0.463	0.094	0.308	0.536	0.54	0.555	0.534	0.482	0.471	0.486	0.099
**OD**	0.089	0.426	0.103	0.309	0.492	0.51	0.491	0.495	0.465	0.526	0.483	0.093
**OD**	0.087	0.462	0.12	0.332	0.526	0.539	0.484	0.483	0.458	0.509	0.492	0.093
** OD**	0.095	0.127	0.111	0.098	0.096	0.121	0.098	0.101	0.085	0.083	0.09	0.096
**Mean Value**	0.092	0.446	0.098	0.314	0.518	0.534	0.509	0.509	0.487	0.486	0.498	0.094

### 3.4. Fifty Percent Tissue Culture Infectious Dose 

Vero cells were cultured in 24-well microplates and inoculated with serial dilutions of HSV-1. The microplates were incubated for one hour in CO_2_ incubator at 37℃. After the incubation period, the wells were washed with PBS and then DMEM medium was added to each well. The microplates were incubated at 37℃ and 5% CO_2_ for seven days and were examined daily for the presence of CPE. Any experiment was examined three times and titers of all preparations were determined by Reed and Muench’s method (10).

### 3.5. Antiviral Assays

Antiviral effects of licorice root extract on HSV-1 was designed and evaluated in three steps that are discussed below.

#### 3.5.1. Incubation of HSV-1 With Licorice Root Extracts prior to HSV-1 inoculation

To determine whether the extract had any antiherpetic activity against the virus particle, virus serial dilutions were prepared by noncytotoxic concentration (0.2 mg/mL) of the licorice root extract and incubated for one hour at 4℃. After the incubation, the virus dilutions were used to infect Vero cells and incubated in CO_2_ incubator at 37℃ for one hour. Following one hour incubation, unabsorbed virus preparation was removed and the cells were washed by PBS and DMEM; then 2% FBS was added to the monolayer cells. The microplates were incubated at 37℃ with 5% CO_2_ and examined seven days for the evidences of CPE.

#### 3.5.2. Pre- Treatment of Vero Cells With Licorice Root Extract Prior to HSV-1 Inoculation

Vero cells were pretreated with nontoxic concentration of the extract (0.2 mg/mL) for two hours at 37℃ with 5% CO_2_. In the next step, the extract was removed and cells were washed by PBS and then inoculate with 100 TCID_50_/mL of HSV-1. The other steps were as described before. The microplates were monitored for seven days to detect any CPE.

#### 3.5.3. Incubation of Vero Cells With Licorice Root Extract After HSV-1 Inoculation

After infecting the monolayer cells with 100 TCID_50_/mL of HSV-1 and one hour incubation, the Vero cells were washed with PBS; then nontoxic concentration of the extract (0.2 mg/mL) in DMEM was added to the monolayer cells after one, four, eight, and twelve hours of viral infection. The 24-well plates were incubated in CO_2_ incubator at 37℃ and monitored daily up to seven days. Viral titer was determined by the endpoint dilution method and calculated TCID_50_ was compared with control virus sample preparation.

### 3.6. Statistical Analysis

The obtained data were analyzed using SPSS v.16 (SPSS Inc., Chicago, IL, USA).One-way ANOVA test was used to compare the quantitative parameters among the study groups. In the cases of any difference among groups, Tukey’s HSD test was utilized to determine the different group. All tests were considered statistically significant if the P values was less than 0.05. 

## 4. Results

### 4.1. Cytotoxicity

Cytotoxicity of the licorice root extract was examined by Neutral Red assay. The mean value of wells assigned to each extract concentration was measured. The highest dilution that had less than 50% cytotoxicity for Vero cells was 1:50, which was equal to a concentration of 0.2 mg/mL. At the mentioned concentration, the Vero cells appeared normal and no noticeable CPE was seen in microscopic monitoring; therefore, the concentrations higher than 0.2 mg/mL were used for further experiments.

### 4.2. Virus Yield Reduction Assay

The possible inhibition of virus yield under the effect of the licorice extract was assessed as TCID_50_/mL in Vero cells in comparison to the virus stock with 105.6 TCID_50_/mL. As described previously, we examined the possible inhibitory effect of the licorice root extract on HSV-1 yield in three steps. The TCID_50_ of the virus in these two experiments reduced about one logarithm in comparison to the control virus, which was significant.

**Figure 2.**
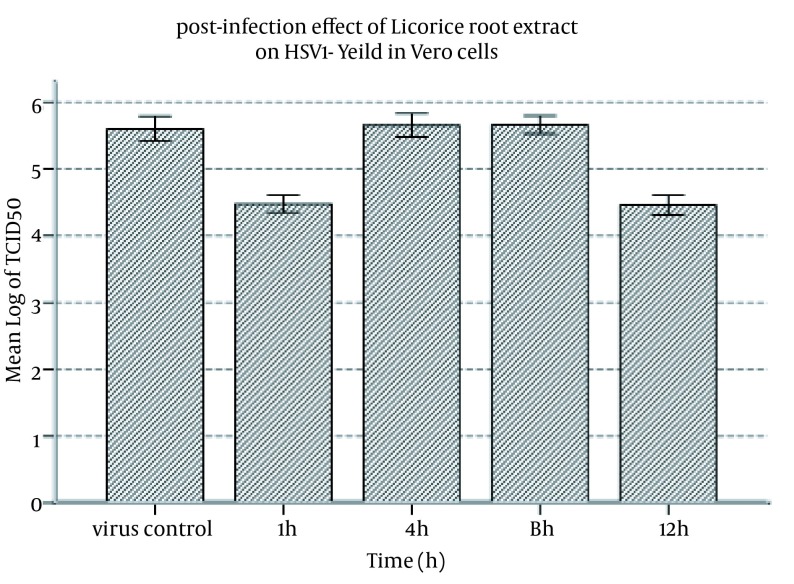
Postinfection Effect of Licorice Root Extract on HSV-1 Yield in Vero Cells. After infecting the monolayer cells with HSV-1 and one-hour incubation period, nontoxic concentration of the extract in DMEM was added to the monolayer cells after one, four, eight, and twelve hours of viral infection. The 24-well plates were incubated in CO_2_ incubator at 37℃ and monitored daily up to seven days. Viral titer was determined by the endpoint dilution method and calculated 50% tissue cellular infectious dose (TCID50) was compared to the control virus sample preparation.

In order to assess the antiherpetic activity of the licorice root extract after virus adsorption, the licorice extract was incubated with Vero cells infected with HSV for one, four, eight, and twelve hours postinfection. The results showed that the effect of the extract was changed with regard to the elapsed time of incubation (F = 309.146; df = 4.10; P < 0.001). Tukey’s HSD test showed similar effect of the elapsed time after four and eight hours incubation of the extract in comparison to the control virus, which were not significant (P = 0.836 and P = 0.805, respectively); however, the effect of the extract was significant after one and 12 hours incubation of extract in comparison to the control virus, which was more than the logarithm variation (P < 0.001) (Table 2). Comparing the internal association among different groups of experiments showed that the different efficacy of the extract with respect to the elapsed incubation time was significant between one and four hours, one and eight hours, four and 12 hours, and eight and 12 hours (change > 1 log) (Table 2 and Figure 2).

**Figure 3.**
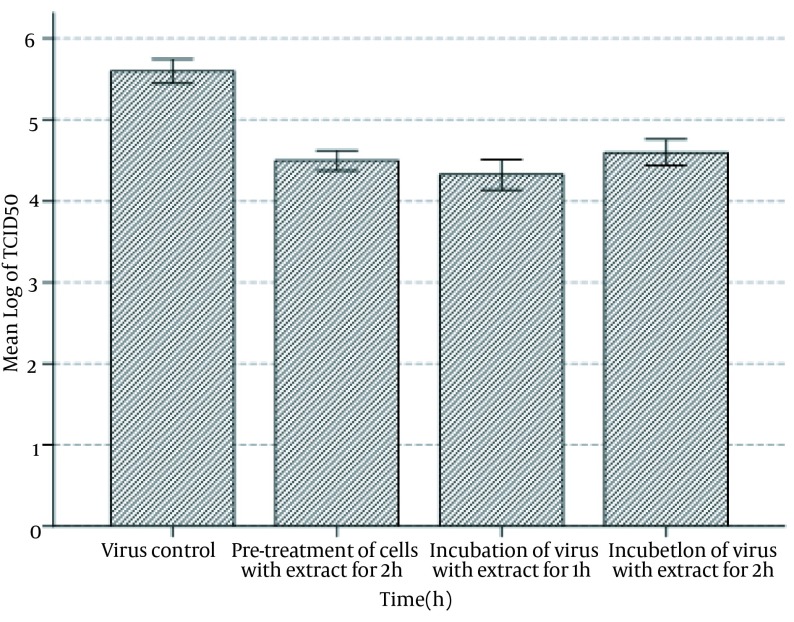
Postinfection Effect of Licorice Root Extract on HSV-1 Yield in Vero Cells Following Pretreatment of Virus Preparation With Licorice Root Extract Vero cells were pretreated with nontoxic concentration of extract for two hours in 37℃ with 5% CO_2_. In the next step, the cells were infected with HSV-1 for one-hour and two-hour incubation period. The Vero cells were washed with PBS and nontoxic concentration of the extract in DMEM was added to the monolayer cells. The microplates were monitored up to seven days for the presence of cytopathic effects. Then calculated 50% tissue cellular infectious dose (TCID_50_) was compared to the control virus sample preparation.

To compare the effect of pretreatment of cells with licorice root extract and preincubation of virus with licorice root extract, new experiments were designed, ie, the results were compared to control virus. Pretreatment of cells with extract for two hours and incubation of virus with extract for one and as well as two hours had significant differences among the study groups (F = 247.412; df = 3.8; P < 0.001). Tukey’s HSD test showed the significant effect of any of the mentioned groups in comparison to the control virus (P < 0.001) (Table 3). Moreover, the comparison between pretreatment of cells with extract for two hours and incubation of virus with extract for one hour showed a significant difference in the antiviral effect of licorice root extract (P < 0.040); however such an effect was not significant in comparison to the incubation of virus with extract for two hours (P = 0.31). Incubation of virus with licorice root extract showed significant difference in antiviral effect of licorice root extract between one and two hours incubation (P < 0.001) (Table 3 and Figure 3).

**Table 2.** Relative Comparison of the Anti-HSV1 Activity of Licorice root Extracts in Different Incubation Time Courses ^a^

Virus Control/Different Time Courses	Different Time Courses	Mean Difference		P value ^b^
**Virus Control**	1 h	1.127 ± 0.052		< 0.001
**Virus Control**	4 h	-0.053 ± 0.052		0.836
**Virus Control**	8 h	-0.057 ± 0.052		0.805
**Virus Control**	12 h	1.147 ± 0.052		< 0.001
**1 h**	4 h	-1.180 ± 0.052		< 0.001
**1 h**	8 h	-1.183 ± 0.052		< 0.001
**1 h**	12 h	0.020 ± 0.052		0.994
**4 h**	8 h	-0.003 ± 0.052		> 0.999
**4 h**	12 h	1.200 ± 0.052		< 0.001
**8 h**	12 h	1.203 ± 0.052		< 0.001

^a^ Data are presented as mean ± standard deviation.

^b^ P value < 0.05 was considered statistically significant.

**Table 3.** Comparison of the Anti-HSV-1 Activity of Licorice Root Extracts in Different Time Courses Following Incubation of Virus With Extract ^a^

Virus Control / Different Time Courses	Different Time Courses	Mean Difference		P Value
**Virus Control**	Pre-treatment of cells with extract for 2 hrs	1.100 ± 0.052		< 0.001
**Virus Control**	Incubation of virus with extract for 1h	1.273 ± 0.052		< 0.001
**Virus Control**	Incubation of virus with extract for 2 hrs	1.003 ± 0.052		< 0.001
**Pretreatment of Cells With Extract for 2 h**	Incubation of virus with extract for 1 h	0.173 ± 0.052		0.040
**Pretreatment of Cells With Extract for 2 h**	Incubation of virus with extract for 2 hrs	-0.097 ± 0.052		0.310
**Incubation of Virus With Extract for 1 h**	Incubation of virus with extract for 2 hrs	-0.270 ± 0.052		< 0.001

^a^Data are presented as mean ± standard deviation.

## 5. Discussion

The results of the current study were almost unique. We found more details concerning antiherpetic activity of licorice root extract including a key observation that determined the efficacy of time course of extract treatment in the cells infected with virus. Thus, this research highlights the importance of the time course in the process of antiviral effect of *G. glabra* aqueous extract. In addition, the role of virus pretreatment with licorice root extract was notable. Moreover, the reliability of TCID_50_ test in determining the suitable concentration of licorice root aqueous extract (0.2 mg/mL), which had less than 50% cytotoxicity for Vero cells, was justified.

The antiherpetic activity of licorice root extract may be due to a number of mechanisms such as the role of *G. glabra* in the inhibition of HSV attachment process through direct contact between virus and the extract. In this situation, the HSV-1 was inhibited by either directly inactivation of virus or antiadhesive property of *G. glabra* aqueous extract, which hampers the adhesion of HSV-1 to Vero, cells in vitro. The latter hypothesis is in agreement with Wittschier et al. findings that confirmed the polysaccharides isolated from the aqueous extract of *G. glabra* roots present so strong antiadhesive property that is able to suppress the adhesion of *Helicobacter pylori* to human gastric mucosa. Wittschier et al. believed that this effect was related to the polysaccharides isolated from the aqueous extract of *G. glabra* (11). Furthermore, glycyrrhizin and glycyrrhizic acid are capable of hampering the growth and CPE of HSV (5, 12).

In addition, based on the results of the current study, it might be speculated that the suppression of HSV-1 replication in Vero cells occurs by interruption of the late stages of genes expression. Data from another study appears to be consistent with the interruption of genes expression in HSV by a medicinal plant named *Chamaecyparis* *obtuse*; however, it was effective in the immediate-early stages of gene expression (13). Moreover, traditional Chinese herb, namely, *Tripterygium hypoglaucum,* has been shown to have anti-HSV properties by suppressing early and late stages of genes expression (14). Other studies have also indicated that licorice root and its constituents, perform an antiviral activity against HSV that permanently inactivates the virus (5, 15, 16). This might be due to the different components of this plant including glycyrrhizic acid, which inactivates *HSV* particles (5). Moreover, animal studies have reported that glycyrrhizin and its derivatives are capable to decrease viral activity and the mortality rate in *HSV* encephalitis (3). Another report revealed the key role of glycyrrhizin in improving the impaired resistance of injured mice to the infection by HSV (17).

Both compounds of licorice root, the triterpene glycoside glycyrrhizic acid (glycyrrhizin) and its aglycone 18-beta-glycyrrhetinic acid, were confirmed to have anti-inflammatory, antioxidant, antitumor, and antiviral properties (18, 19). In addition, the antiviral effects of glycyrrhizin by suppressing replication of several viruses were shown in vitro (20, 21). Glycyrrhizin also provides a plausible mechanism for a broad spectrum of antiviral activities including HSV, *Flavivirus*, *Human immunodeficiency virus*, *Vaccinia virus*, *Poliovirus* (type 1), *Vesicular stomatitis virus*, IAV, SARS-related *Coronavirus*, *human* *respiratory syncytial virus*, and Arboviruses (3, 5, 22-29).

Future researches are needed to investigate the effect of methanol extraction of *G. glabra* on HSV-1. Comparing the results can help us to identify the possible correlation or differences in physiologic effects between these two different extracts. Moreover, further in vivo studies should be conducted to identify significant possible side effects or cytotoxicity of *G. glabra* in animal models and high risk people such as pregnant women, the elderly with heart disease, asthma, etc. This will help us to verify the inappropriate effects of *G. glabra* applications in human.

In conclusion, although *G. glabra* showed the characteristics of a novel antiviral medication, more in vitro experiments are need to declare the role of chemically derivatives of *G. glabra,* which may present a broad range of antiherpetic activities.
